# Ambient and household air pollution on early-life determinants of stunting—a systematic review and meta-analysis

**DOI:** 10.1007/s11356-021-13719-7

**Published:** 2021-04-09

**Authors:** Vivian C. Pun, Russell Dowling, Sumi Mehta

**Affiliations:** 1 Environmental Health Division, Vital Strategies, Singapore office: 6A Shenton Way, OUE Downtown, #04-01, Singapore, 068815 Singapore; 2 Environmental Health Division, Vital Strategies, New York office: 100 Broadway, 4th Floor, New York, NY 10005 USA

**Keywords:** Ambient air pollution, Household air pollution, Stunting, Height-for-age z-score, Small for gestational age

## Abstract

Stunting is an important risk factor for early growth and health implications throughout the life course, yet until recently, studies have rarely focused on populations exposed to high levels of particulate matter pollution or on developing countries most vulnerable to stunting and its associated health and developmental impacts. We systematically searched for epidemiologic studies published up to 15 August 2020 that examined the association between ambient and household particulate exposure and postnatal stunting (height-for-age z-score) and prenatal determinants (small for gestational age or SGA, or equivalent) of stunting. We conducted the literature search in PUBMED, MEDLINE, EMBASE, and Web of Science databases in August 2020, using keywords including, but not limited to, “particulate matter,” “indoor/household air pollution,” and “adverse birth outcomes,” to identify relevant articles. Forty-five studies conducted in 29 countries met our inclusion criteria for meta-analysis. We found significant positive associations between SGA and a 10 μg/m^3^ increase in fine particulate matter (PM_2.5_) exposure over the entire pregnancy [OR = 1.08; 95% confidence interval (CI): 1.03–1.13], with similar SGA impact during the second and third trimesters, and from high exposure quartile of PM_2.5_ exposure during the entire pregnancy. A 19% increased risk of postnatal stunting (95% CI: 1.10, 1.29) was also associated with postnatal exposure to household air pollution. Our analysis shows consistent, significant, and noteworthy evidence of elevated risk of stunting-related health outcomes with ambient PM_2.5_ and household air pollution exposure. This evidence reinforces the importance of promoting clean air as part of an integrated approach to preventing stunting.

**Supplementary Information:**

The online version contains supplementary material available at 10.1007/s11356-021-13719-7.

## Background

Air pollution is a major environmental health threat and an important determinant of child health globally. The World Health Organization (WHO) recognizes air pollution as “an overlooked health emergency for children around the world,” noting that the issue can be especially severe for children living in low- or middle-income countries (LMICs) (WHO 2018). Exposure to air pollution was the second leading risk factor for premature death among children under 5 in 2019, resulting in over 691,000 deaths, two-thirds of which was from household air pollution exposure (IHME 2021). Unlike many more prominent risk factors, air pollution is pervasive—93% of children and teens ≤ 15 years globally are exposed to ambient PM_2.5_ levels higher than 10 μg/m^3^ (WHO 2018), the health-based limit in WHO’s Air Quality Guidelines. Burning, or combustion, is the main source of health-damaging air pollutants, including particulate matter less than 2.5 microns in diameter (PM_2.5_). One specific combustion source— cooking or heating with dirty fuels—can result in smoky indoor concentrations of PM_2.5_ and other pollutants five or six times the levels in ambient air, posing significant health risks to women and children in these households while contributing substantially to ambient air pollution levels (WHO 2018). While PM_2.5_ at high concentrations is visible as smoke or haze, even concentrations too low to be visible can have health effects.

Exposure to elevated levels of particulate pollution during childhood is associated with increases in pneumonia, asthma, bronchitis, and other respiratory infections and diseases (UNICEF 2016a, 2016b). In recent years, many researchers have turned to elucidating the relationship between in utero exposures and adverse pregnancy outcomes, including low birthweight and preterm delivery, as well impacts on child growth in childhood.

Stunting, or linear growth failure in childhood, refers to a child who is too short for his or her age. Stunting begins in utero and manifests mostly during the first 2 years of postnatal life, with increasing prevalence until age 5 (WHO 2013; Prendergast and Humphrey 2014; Goudet et al. 2015). Stunting is a largely irreversible outcome with long-term impacts on children and their communities. In addition to height and physical development concerns, stunted children often achieve lower developmental test scores and suffer from diminished cognitive development and reduced economic activity (de Onis and Branca 2016; Hoddinott et al. 2015; UNICEF/WHO/World Bank 2019; UNICEF 2016b; Victora et al. 2010; Woldehanna et al. 2017). Globally, an estimated 144 million (21%) children under 5 in 2019 were stunted, with a height-for-age z-score (HAZ) of − 2 standard deviations (SD) below the WHO child growth standards median (UNICEF/WHO/World Bank 2020). The highest prevalence of stunting is in Oceania (38%), followed by Africa (29%) and Asia (22%).

### Study Purpose and Rationale

Early epidemiologic studies have found that children who lived in areas and households with polluted air exhibited shorter height stature than those with cleaner air. Bobak et al. (2004), for example, observed up to a 1.2-cm difference in height between children living in the most polluted communities (particulate level of 281 μg/m^3^) and those in the cleanest communities (67 μg/m^3^). Two previous meta-analyses have summarized the earlier air pollution studies on prenatal determinants of stunting, defined as small for gestational age (SGA) or intrauterine growth restriction (IUGR) (Zhu et al. 2015), and postnatal stunting measured by HAZ (Bruce et al. 2013). Collectively, these studies suggested positive associations between air pollution and stunting. However, a significant number of recently published studies not included in the previous meta-analyses have yielded results that address key gaps in the evidence base of the air pollution-stunting association, especially regarding the strength of evidence for household air pollution or ambient pollution concentrations several-fold greater than the WHO air quality guidelines, both of which occur in regions with the highest stunting prevalence. We therefore aimed to provide an updated systematic review and meta-analysis of the evidence of the association of ambient and household air pollution on stunting-related outcomes. Specifically, we reviewed all studies that evaluate the impact of air pollution on postnatal stunting (i.e., HAZ) among children aged 5 or under, as well as adverse pregnancy outcomes (i.e., SGA, IUGR) that also serve as strong prenatal determinants of stunting. Previous studies have shown SGA to be highly associated with postnatal stunting (e.g., 3.55 odds of stunting among SGA newborns) (Christian et al. 2013; Xie et al. 2016).

## Methods

### Search strategy

A systematic search of peer-reviewed articles that assessed the effect of ambient and/or household particulate matter exposure during or post-pregnancy on stunting-related outcomes was carried out on PUBMED, MEDLINE, EMBASE, and Web of Science databases in August 2020. The following terms were used in the search:

- Exposures: “air pollution” or “particulate matter,” “PM_2.5_,” “PM_10_,” “indoor air pollution,” “household air pollution,” “solid fuel,” “biomass” or “cooking”
- Outcomes: “adverse pregnancy outcomes,” “adverse birth outcomes,” “stunting,” “height-for-age” (“HAZ”), “length-for-age” (“LAZ”), “small for gestational age” (“SGA”), “intrauterine growth restriction” (“IUGR”)

All original studies published between 1999 and 2020 (up to 15 August 2020) were considered. Reference lists of previous relevant published reviews, meta-analyses, and the identified articles were also searched. The last search was updated on 17 August 2020. Daily time-series studies, case reports, case studies, and studies available only in abstract form were excluded. All studies were evaluated independently by two reviewers to ensure identified articles were suitable for inclusion in the meta-analysis. Reporting of findings of this systematic review was guided by the Preferred Reporting Items for Systematic Reviews and Meta-Analyses (PRISMA) checklist and flow diagram (Moher et al. 2010).

### Eligibility criteria and study selection

Eligible studies included in the meta-analysis met all the following inclusion criteria:

- Population: studies of pregnant women and their newborns and studies of children (≤ 5 years of age)
- Original articles examining exposure to particulate matter with an aerodynamic diameter less than 10 μm (PM_10_), PM_2.5_, or household air pollution exposure from cooking with solid fuel;
- Definitions of growth faltering: stunting defined as HAZ < − 2 standard deviation (SD) or LAZ < − 2 SD, and severe stunting defined as HAZ < − 3 SD of the WHO child growth standards or reference population median; SGA defined as birth weight below the 10th percentile for a given gestational age and gender of the newborn in the study population or similar; or IUGR defined as birth weight below the 10th percentile for gestational age or similar;
- Quantitative evaluation (i.e., linear or logistic regression coefficients) of the relationship;
- Exposure period was calculated as whole pregnancy and/or specific trimesters;
- Articles were written in English.

Studies or effect estimates were excluded if one or more of the following occurred:

- Did not meet at least one of the above inclusion criteria;
- Exposure period (e.g., trimester-specific, during or post-pregnancy) was not explicitly reported;
- Source-specific particulate pollution (e.g., PM_2.5_ from incinerator or vehicle exhaust);
- Effect estimates in studies could not be converted into odds ratio (OR) and 95% confidence intervals (CI) in risk of stunting;
- Studies adjusted for two or more pollutants in the same model;
- Reviews and repeat literature including secondary analysis of data.

For multiple publications of the same birth population in a region of the same outcome, only the study with the largest number of observations and/or the longest study period was included in the meta-analysis to avoid the overlapping datasets in the same outcome. Likewise, if multiple estimates using various exposure assessment tools (e.g., monitoring, satellite) of the same outcome from the same population were presented in a single study, only estimates with the largest sample size and/or smallest standard errors were included.

### Data extraction and statistical analysis

Information extracted from each study included first author’s surname, year of publication, country, study period, birth population, birth outcomes, exposure type (e.g., PM_2.5_, solid fuel), exposure assessment (e.g., satellite, monitoring data), exposure period (e.g., trimester specific and/or entire pregnancy), risk measure (e.g., odds ratio, relative risk), effect size, and adjustment of other factors or covariates. As all eligible studies that examined ambient particulate pollution reported effect estimates either for continuous exposure (when they assumed linear associations) or by categories (e.g., tertiles, quartiles, or quintiles) of exposure, we performed all analyses using both continuous exposure (per 10 μg/m^3^ increase in PM_2.5_ or PM_10_ concentration) and categorical exposure defined as high vs. low, where the high level was defined as the highest study-specific category and the low level as the lowest study-specific category. For household particulate pollution, analysis was performed using categorical exposure defined as high vs low exposure. To ensure comparability, reported relative risks were converted to ORs using the approximation approach described by Zhang and Yu (Zhang and Yu 1998), when the incidence of an outcome of interest was < 10% in the study population or if the information on cases and non-cases were available from the study.

Given that SGA and IUGR are often used interchangeably, both outcomes were analyzed together as SGA in our study. Studies with LAZ as outcome were also analyzed as HAZ. A random-effects model was used to estimate the pooled effect measures, with between-study heterogeneity assessed using *I*^2^ statistic (25%, 50%, and 75% were used as rules of thumb for low, moderate, and high heterogeneity, respectively) (Higgins and Thompson 2002). In this meta-analysis, effect estimates were grouped by exposure period (e.g., trimester specific), and heterogeneities were also analyzed by exposure period. Possible publication bias was assessed with Begg’s test and the degree of asymmetry was evaluated by Egger’s tests (Begg and Mazumdar 1994; Egger et al. 1997). As part of the sensitivity analysis, each eligible study was removed one-by-one from the meta-analysis to examine the robustness of our pooled ORs. We restricted the analysis to eligible studies that had the same outcome definitions. All statistical analyses were performed using R Software version 4.0.2 (R Foundation for Statistical Computing, Vienna, Austria), and a *p*-value < 0.05 is considered statistically significant.

## Results

### Characteristics of included studies

**Fig. 1:**
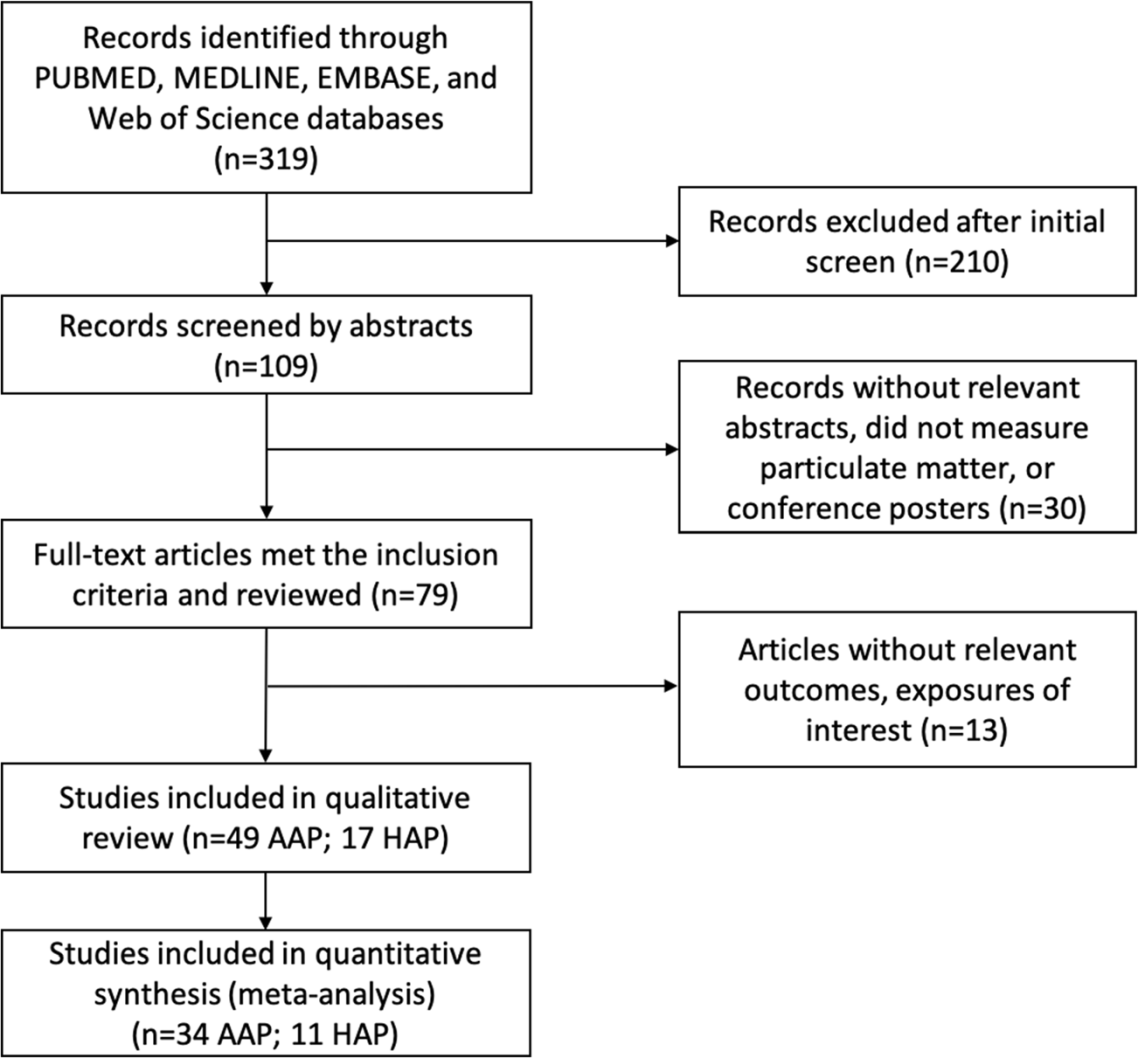
Flow diagram of included/excluded studies (AAP, ambient air pollution; HAP, household air pollution)

Figure 1 shows the study screening process. Briefly, of the 319 articles identified in the literature search and reference lists of previous relevant reviews, 109 articles with possibly eligible titles were selected by either reviewer, and 30 were subsequently excluded because of irrelevant abstracts. Full manuscripts of 79 articles were reviewed, with thirteen further excluded. Overall, 66 articles were included in the systematic review (49 for ambient and 17 for household air pollution). The number of studies published increases gradually since 1999, with 13 articles published in 2019 alone (Figure S1). Of the 49 studies on ambient air pollution, the population of participants from individual studies ranged from 790 to 3 million. The prevalence of SGA ranged from < 1 to 16%, whereas PM_2.5_ and PM_10_ exposures ranged from 4–81 μg/m^3^ and 12–116 μg/m^3^, respectively. Exposure assessment methods applied in most studies were based on existing central monitoring data, while a few used advanced monitoring approaches (e.g., satellite data or modeled pollution measurement from land-use regression models). Most (*n* = 34) were from North America, Europe, and Oceania where ambient air pollution concentrations were low (average PM_2.5_ level: 11 μg/m^3^). Fourteen studies were from Asia (average PM_2.5_ level: 61 μg/m^3^), and one was from South America. Conversely, most studies on household air pollution included in the meta-analysis were from Asia (*n* = 11) and Africa (*n* = 2) where a high proportion of the population uses solid fuels. One study contained data from seven developing countries across the globe (Kyu et al. 2009). The number of participants from individual studies ranged from 112 to nearly 207,000. The prevalence of stunting ranged from 8 to 89%, whereas the percentage of the population using solid fuels in countries from those studies ranged from 11 to 97%. Overall, covariate adjustments varied considerably though key adjustments included maternal age, maternal education, parity, and infant sex. Twenty-six studies adjusted for socioeconomic status and 19 studies adjusted for tobacco use.

Of the articles included in the systematic review, forty-five articles were included in the meta-analysis (Table S1 summarizes the characteristics of the included studies). Among them, 34 studies examined either SGA or IUGR and ambient PM_2.5_ or PM_10_ pollution exposure during the entire pregnancy and/or any of the three trimesters, and 11 examined the impact of post-pregnancy exposure on a dichotomous measure of HAZ among children aged 5 or under. Studies excluded from the meta-analysis were not considered due to an insufficient number of effect estimates in the same exposure/outcome groupings (e.g., SGA or IUGR with different definitions than 10%).

### Pooled estimates of ambient air pollution

**Fig. 2:**
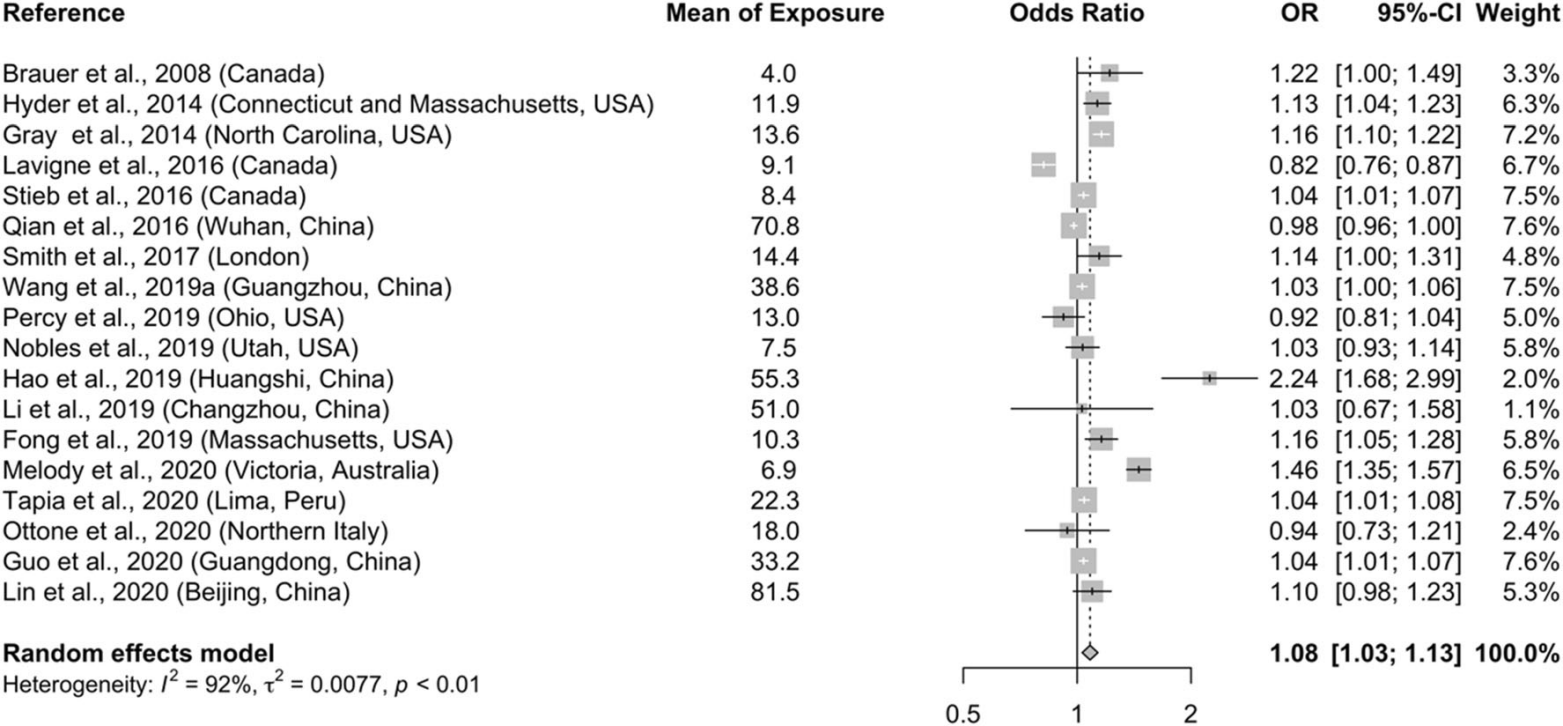
Pooled estimates of the effect on SGA associated with a 10 μg/m^3^ increase in PM_2.5_ concentration during the entire pregnancy

**Fig. 3:**
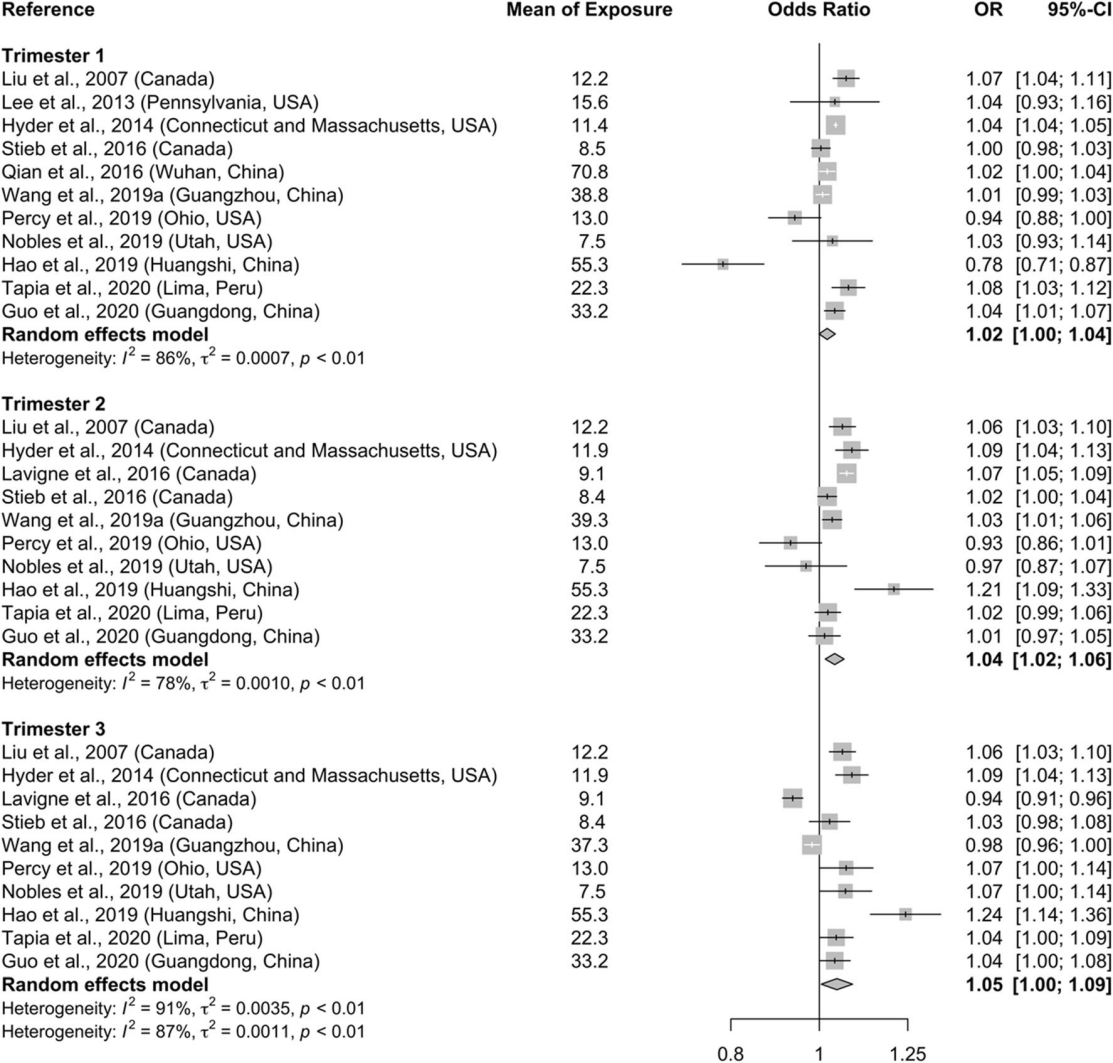
Pooled estimates of the effect on SGA associated with a 10 μg/m^3^ increase in PM_2.5_ concentration during three trimesters

Table 1 summarizes the pooled effect estimates of air pollution on adverse pregnancy outcomes. We found that a 10 μg/m^3^ increase in PM_2.5_ during the entire pregnancy (OR = 1.08; 95% CI: 1.03–1.13) was significantly associated with SGA (Fig. 2), as well as elevation in particulate pollution during second (OR = 1.04; 95% CI: 1.02–1.06) and third trimesters (OR = 1.05; 95% CI: 1.00–1.09; Fig. 3). Increased pooled ORs were also observed for the first trimester and for SGA risks of high versus low quartiles of PM_2.5_ exposure during the entire pregnancy, though the associations were not statistically significant at the alpha level of 0.05. The slightly stronger magnitude of the associations (e.g., OR = 1.09; 95% CI: 1.04–1.14 for entire pregnancy; Figure S2) was observed when we included a few studies that were used in the past published meta-analysis but were excluded from our main analysis because of less stringent SGA definitions. In addition, we found significant elevated odds of SGA associated with a 10 μg/m^3^ increase in PM_10_ level during entire pregnancy (OR = 1.03; 95% CI: 1.00–1.05) and first trimester (OR = 1.01; 95% CI: 1.00–1.03; Figure S3), as well as with high quartile of PM_10_ exposure (OR = 1.10; 95% CI: 1.02–1.18).

**Table 1:** Summary of combined effect estimates on stunting associated with particulate exposures

Outcome and PM type	Exposure measure	Trimester	No. of studies	Test of associations (random effect)		Test of heterogeneity		Publication bias	
				OR [95% CI]	*p*-value	*I*^2^ (%)	*p*-value	Egger’s test (*p*-value)	Begg’s test (*p*-value)
Ambient air pollution									
SGA and PM_2.5_	Continuous (per 10 μg/m^3^)	Entire pregnancy	18	**1.08 [1.03, 1.13]**	**0.0013**	92%	< 0.0001	0.1294	0.4264
		Trimester 1	11	1.02 [1.00, 1.04]	0.0589	86%	< 0.0001	0.1122	0.5858
		Trimester 2	10	**1.04 [1.02, 1.06]**	**0.0014**	78%	< 0.0001	0.5287	0.7884
		Trimester 3	10	**1.05 [1.00. 1.09]**	**0.0282**	91%	< 0.0001	0.0106	0.0603
	Categorical (high vs low)	Entire pregnancy	5	1.05 [0.99, 1.12]	0.1136	0.0%	0.6413	0.1221	0.1416
SGA and PM_10_	Continuous (per 10 μg/m^3^)	Entire pregnancy	12	**1.03 [1.00, 1.05]**	**0.0361**	87%	< 0.0001	0.0665	0.0549
		Trimester 1	11	**1.01 [1.00, 1.03]**	**0.1059**	85%	< 0.0001	0.6521	0.4835
		Trimester 2	7	1.02 [1.00, 1.04]	0.0768	85%	< 0.0001	0.4679	0.5312
		Trimester 3	9	1.02 [1.00, 1.04]	0.0901	86%	< 0.0001	0.4267	0.4042
	Categorical (high vs low)	Entire pregnancy	5	**1.10 [1.02, 1.18]**	**0.0146**	35%	0.1854	0.2672	0.3272
Household air pollution									
HAZ < − 2 SD*	Use of solid fuel (high vs. low)	Post pregnancy	11	**1.19 [1.10, 1.29]**	**< 0.0001**	55%	0.0128	0.5593	0.5858
HAZ < − 3 SD		Post pregnancy	3	1.12 [1.00, 1.26]	0.0548	18%	0.2953	0.3265	0.6015

*SD refers to standard deviation. Bold font indicates statistical significance at alpha = 0.05 level

Overall, study heterogeneity was estimated in random-effect models. We observed a high degree of heterogeneity (*I*^2^ ≥ 85%) among studies that used continuous measure of PM_2.5_ and PM_10_ as exposure metric (e.g., SGA and PM_2.5_ for entire pregnancy, *I*^2^ = 92%, *p* < 0.001) but not for studies that reported findings for categorical measures of PM_2.5_ and PM_10_ (PM_2.5_, *I*^2^ = 0%, *p* = 0.6413; PM_10_, *I*^2^ = 35%, *p* = 0.1854). Our pooled estimates were robust. Removing a particular study did not affect the pooled estimates by more than 3% (data not shown), and the significance of the associations did not change except in a few instances. That is, the observed association between SGA and PM_2.5_ exposure during the first trimester became statistically significant when either Percy et al. (2019) or Hao et al. (2019) was removed; on the other hand, an association for SGA and PM_2.5_ exposure during the third trimester was no longer significant after either Liu et al. (2007), Hyder et al. (2014), or Hao et al. (2019) was removed. Nonetheless, there was no significant publication bias for all exposures and outcomes except for the association between SGA (continuous) PM_2.5_ exposure during the third trimester (Egger’s test *p* < 0.05).

### Pooled estimates of household air pollution

**Fig. 4:**
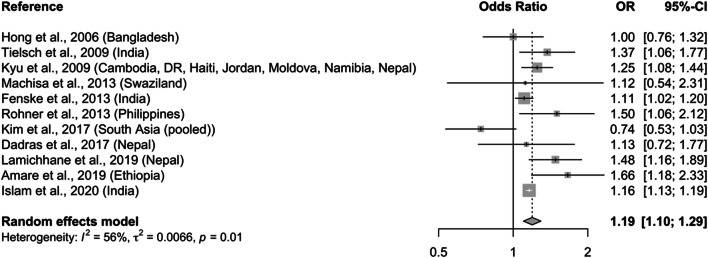
Pooled estimates of the effect on stunting (HAZ < − 2) among children < 5 years old associated with exposure to household air pollution from solid fuel use compared with cleaner fuels

Two pooled estimates of the effect of stunting (i.e., HAZ < − 2 SD) and severe stunting (i.e., HAZ < − 3 SD) associated with household exposure to solid fuels were calculated. Pooled OR for stunting from eleven epidemiologic studies is 1.19 (95% CI: 1.10–1.29; Fig. 4), with moderate heterogeneity (*I*^2^ = 55%; *p* = 0.01) and no publication bias (Table 1). The effect estimate was robust in sensitivity analysis upon removing one study (data not shown). For severe stunting only, the pooled estimate is 1.12 (95% CI: 1.00–1.26; Figure S4), with no evidence of statistical heterogeneity (*I*^2^ = 18%; *p* = 0.2953) nor publication bias.

## Discussion

In this comprehensive quantitative analysis, we summarized the most up-to-date evidence from 45 epidemiologic studies published on or before mid-August 2020 and found ambient and household air pollution exposures were linked to significant and noteworthy increases in the risk of stunting (e.g., HAZ) and prenatal determinant of stunting (e.g., SGA). A 10 μg/m^3^ increase in PM_2.5_ levels over the entire pregnancy was associated with an 8% (95% CI: 3–13%) increase in the risk of SGA, and a range of 2%–5% elevated risk was observed with increased PM_2.5_ exposure during the three trimester periods. Similarly, exposure to household air pollution was associated with a 19% (95% CI: 10–29%) increase in the risk of stunting among children aged 5 or below, with suggestive positive associations observed for severe stunting.

We identified three previous systematic reviews linking ambient air pollution with SGA and IUGR (Zhu et al. 2015; Yuan et al. 2019) and household air pollution with HAZ (Bruce et al. 2013). Of the reviews that focused on ambient PM_2.5_ exposure, only Zhu et al. (2015) conducted a quantitative evaluation of the evidence, while Yuan et al. (2019) only offered qualitative observations. In a 2015 review, Zhu et al. (2015) evaluated six studies on the association between PM_2.5_ and SGA and reported OR of 1.15 (95% CI: 1.10–1.20) for SGA per increase in PM_2.5_ exposure during the entire pregnancy. When we included the same studies and 12 additional new publications in our latest meta-analysis, we found an OR of 1.09 (95% CI: 1.04–1.14; Figure S2). Our pooled OR estimates are smaller in magnitude than those reported in Zhu et al. (2015). One explanation for this is that individual epidemiologic studies published after the 2015 meta-analysis tend to report effect estimates that are lower in magnitude and statistical significance compared to earlier studies. More recently published studies were from cities in Asia (e.g., China), where PM_2.5_ levels were much higher than in cities with much lower pollution levels (i.e., 4–22μg/m^3^) where earlier studies were conducted.

This phenomena of decreasing effect size with increasing PM_2.5_ concentrations have been previously documented (Vodonos et al. 2018) and suggests a non-linear association between exposure and response. Nonetheless, our findings are supported by existing literature on the impact of ambient air pollution on stunting defined as HAZ. A recent study found that prenatal exposure to the 1997 Indonesian forest fires is associated with a 0.41 in HAZ (or 3.4 cm) at age 17, which implies a loss of 4% of average monthly wages for approximately 1 million Indonesian workers born during this period (Tan-Soo and Pattanayak 2019). Results of a study conducted in Bangladesh, where stunting prevalence is as high as 36%, children with a high quartile of PM_2.5_ exposure (52–73 μg/m^3^) had 1.13 times the risk of stunting (HAZ < − 2) than that of children in the lowest quartile of exposure (Goyal and Canning 2018).

For household air pollution, we identified eleven epidemiologic studies for stunting defined by HAZ, including three studies published after a previous meta-analysis (Bruce et al. 2013). Bruce et al. (2013) reported a pooled OR of 1.27 (95% CI: 1.12–1.43) based on two studies. Our pooled effect estimates for household air pollution from cooking with solid fuels are consistent with but smaller than the Bruce et al. (2013) estimates. Overall, the volume of new evidence from both ambient and household particulate exposure suggests a high level of consistency regarding the association between ambient and household particulate pollution and stunting and prenatal determinants of stunting.

There is a strong biological basis for the relationship between air pollution exposure and low birthweight. Kannan et al. (2006) reviewed the evidence from existing literature and determined there are potentially five distinct mechanisms at work: oxidative stress, inflammation, coagulation, endothelial function, and hemodynamic responses. While precise biological mechanisms connecting air pollution with impaired fetal growth are unknown, it is commonly hypothesized that transplacental and postnatal exposure to particulate matter may result in oxidative stress leading to DNA damage. Induced acute placental and pulmonary inflammation, increased possibility for coagulation, and triggered endothelial dysfunction are also hypothesized biological mechanisms.

We acknowledge several limitations in this meta-analysis. First, we observed a moderate to high degree of heterogeneity across gestational exposure periods and exposure metrics. Such heterogeneity may be explained by differences in study design methods and exposure assessment, covariate adjustment, study population, and/or PM chemical composition. Unfortunately, the studies did not provide enough detailed data to explore potential effect modification by socioeconomic status. More evidence on the independent and joint effects of early life exposures to air pollution, nutrition, social class, and the effect of PM constituents on stunting-related risks is warranted. There were not enough studies using other exposure windows (e.g., months) to be evaluated. As a result, our observed effects on stunting-related risks, limited to the entire pregnancy period and specific trimester periods, does not enable us to evaluate the impact of increased exposures to air pollution over shorter time windows. Moreover, while the majority of the included studies evaluated stunting-related outcomes as categorical measures (e.g., HAZ < − 2 SD), it is important to note that growth faltering occurs along the gradient, and children above the conventional cutoff points for SGA/IUGR/HAZ may still experience suboptimal linear growth, especially in low-resource settings (Prendergast and Humphrey 2014; de Onis and Branca 2016). As a result, the actual impact of air pollution on stunting or growth failure may be underestimated. Finally, while we used clear criteria for the inclusion of studies, we did not assess the quality of each included study.

These limitations are balanced by the substantial strengths of our study. Our updated meta-analysis review is timely and warranted given that substantial new evidence has been published since the previous meta-analyses on this topic (6 versus 18 for ambient PM_2.5_ pollution in the current review, 2 versus 11 for household air pollution). Additional value is also derived from updating results of earlier reviews using comparable methods, including effects from both the continuous and categorical measures of PM_2.5_ and PM_10_ exposures. Prior to this review, an effort had not been attempted to join all available childhood stunting-related outcomes or to include a focus on exposures from ambient particulate pollution exposure and household air pollution. Our pooled estimates were robust in sensitivity analyses, as demonstrated by removing one study from the main analysis. There was also no significant publication bias according to Begg’s and Egger’s tests.

## Conclusions

Based on a meta-analysis of 45 eligible studies on the association of air pollution and stunting-related outcomes, we found new evidence demonstrating an increased risk of prenatal determinants of stunting associated with ambient PM_2.5_ exposure during pregnancy (entire pregnancy, and second and third trimester), as well as a significant elevated stunting risk associated with postnatal exposure to household air pollution. Studies included in the analysis reflect a much greater range of exposures than those examined in the previous systematic reviews and also include studies conducted in countries where both adverse pregnancy outcomes and stunting are major public health concerns. Findings suggest the public health relevance of promoting clean air as part of an integrated approach to preventing stunting. For example, in India, a country with one of the highest stunting prevalence (38%) among children under 5 and the highest SGA prevalence (37%) among live births (Lee et al. 2017; UNICEF/WHO/World Bank 2020), reducing the annual mean PM_2.5_ concentration from 91 μg/m^3^ in 2017 to India’s National Ambient Air Quality Standards (40 μg/m^3^) would result in 3 million (or 33%) fewer children born SGA in India, and nearly 4.3 million (or 47%) fewer for reducing to WHO air quality guideline levels (10 μg/m^3^). As such, the integrated evidence presented here provides an additional public health imperative to improve air quality to promote child health and well-being.

## Supplementary information

ESM 1 (DOCX 1.06 mb)

## Data Availability

All data generated or analyzed during this study are included in this published article.

## Abbreviations

CI: confidence interval
HAZ: height-for-age z-score
IUGR: intrauterine growth restriction;
OR: odds ratio
PM: particulate matter
PM_10_: particulate matter with an aerodynamic diameter less than 10 μm
PM_2.5_: particulate matter less than 2.5 μm in diameter
SD: standard deviations
SGA: small for gestational age
WHO: World Health Organization
