# Supplementary material for: Ambient and household air pollution on early-life determinants of stunting—a systematic review and meta-analysis
Source: Environ Sci Pollut Res Int. 2021 Apr 9;28(21):26404–12. doi: 10.1007/s11356-021-13719-7 (PMC8159793; doi:10.1007/s11356-021-13719-7)

**Ambient particulate matter and household air pollution on early-life determinants of stunting – a systematic review and meta-analysis**

Vivian C. Pun; Russell Dowling, Sumi Mehta

**Supplementary**

**Figure S1.** Number of publications on the association between ambient particulate pollution and household air pollution and stunting between 1999 and 2020.

**Table S1.** Characteristics of the studies included in the meta-analysis.

| **Article** | **Data Year** | **Country** | **Study design** | **Sample Size** | **Outcome** | **Outcome prevalence** | **Exposure Period** | **Exposure Measure** | **SES adjusted?** | **Smoking adjusted?** |
| --- | --- | --- | --- | --- | --- | --- | --- | --- | --- | --- |
| ***Ambient PM2.5 pollution*** | | | | | | | | | | |
| Melody et al., 2020 | 2012-2015 | Victoria, Australia | Retrospective cohort study | 285,594 | SGA | 8.9% | Entire pregnancy | PM2.5 | Yes | No |
| Huang et al., 2020 | 1996-2002 | Taiwan | Retrospective cohort study | 322,513 | SGA | 6.9% | Trimesters | PM10 | Yes | No |
| Tapia et al., 2020 | 2012-2016 | Lima, Peru | Retrospective cohort study | 123,028 | SGA | 5.7% | Entire pregnancy, trimesters | PM2.5 | Yes | No |
| Ottone et al., 2020 | 2012-2014 | Northern Italy | Retrospective cohort study | 23,708 | SGA | 5.7% | Entire pregnancy | PM2.5 | Yes | Yes |
| Guo et al., 2020 | 2014-2017 | Guangdong, China | Retrospective cohort study | 2,567,457 | SGA | 13.7% | Entire pregnancy, trimesters | PM2.5, PM10 | No | Yes |
| Lin et al., 2020 | 2014-2017 | Beijing, China | Prospective cohort study | 18,863 | SGA | 5.7% | Entire pregnancy | PM2.5, PM10 | Yes | No |
| Wang et al., 2019a | 2015-2017 | Guangzhou, China | Retrospective cohort study | 506,000 | SGA | 9.7% | Entire pregnancy, trimesters | PM2.5, PM10 | No | No |
| Percy et al., 2019 | 2007-2010 | Ohio, USA | Retrospective cohort study | 224,921 | SGA | 10.8% | Entire pregnancy, trimesters | PM2.5 | No | Yes |
| Nobles et al., 2019 | 2002-2010 | Utah, USA | Retrospective cohort study | 50,005 | SGA | 7.9% | Entire pregnancy, trimesters | PM2.5, PM10 | No | Yes |
| Hao et al., 2019 | 2017 | Huangshi, China | Cross-sectional study | 4,194 | SGA | 7.5% | Entire pregnancy, trimesters | PM2.5, PM10 | No | No |
| Choe et al., 2019a,b | 2002-2012 | Seoul, Korea | Retrospective cohort study | 824,011 | SGA | 11.2% | Entire pregnancy, trimesters | PM10 | Yes | No |
| Li et al., 2019 | 2013-2016 | Changzhou, China | Retrospective cohort study | 2,709 | SGA | 16.2% | Entire pregnancy | PM2.5 | No | No |
| Wang et al., 2019b | 2005-2012 | Shenzhen, China | Cross-sectional study | 1,206,158 | SGA | 7.8% | Entire pregnancy, trimesters | PM10 | Yes | No |
| Fong et al., 2019 | 2001-2012 | Massachusetts, USA | Retrospective cohort study | 725,919 | SGA | 9.1% | Entire pregnancy | PM2.5 | Yes | No |
| Smith et al., 2017 | 2006-2010 | London | Retrospective cohort study | 540,365 | SGA | 9.5% | Entire pregnancy | PM2.5, PM10 | Yes | Yes |
| Capobussi et al., 2016 | 2005-2012 | Italy | Retrospective cohort study | 27,128 | SGA | 10.2% | Entire pregnancy, trimesters | PM10 | Yes | No |
| Lavigne et al., 2016 | 2005-2012 | Canada | Retrospective cohort study | 818,400 | SGA | 9.3% | Entire pregnancy, trimester 2, trimester 3 | PM2.5 | Yes | Yes |
| Stieb et al., 2016 | 1999-2008 | Canada | Retrospective cohort study | 2,966,705 | SGA | 8.3% | Entire pregnancy, trimesters | PM2.5 | No | No |
| Pereira et al., 2016 | 1988-2008 | Connecticut, USA | Retrospective cohort study | 9,587 | SGA | na | Entire pregnancy, trimester 2 | PM10 | No | Yes |
| Qian et al., 2016 | 2010-2013 | Wuhan, China | Prospective cohort study/case-control study | 95,911 | IUGR | 8.8% | Entire pregnancy, trimester 1 | PM2.5, PM10 | Yes | Yes |
| Bijnens et al., 2016 | 2002-2013 | Belgium | Retrospective cohort study | 4,760 | SGA | 9.0% | Entire pregnancy, trimesters | PM10 | Yes | No |
| Winckelmans et al., 2015 | 1999-2009 | Belgium | Retrospective cohort study | 494,653 | SGA | 9.4% | Entire pregnancy, trimesters | PM10 | No | No |
| Hannam et al., 2014 | 2004-2008 | UK | Retrospective cohort study | 37,862 | SGA | 8.6% | Entire pregnancy | PM2.5 | Yes | Yes |
| Hyder et al., 2014 | 2000-2006 | Connecticut and Massachusetts, USA | Retrospective cohort study | 643,839 | SGA | 10.0% | Entire pregnancy, trimesters | PM2.5 | No | Yes |
| Gray et al., 2014 | 2002-2006 | North Carolina, USA | Retrospective cohort study | 457,642 | SGA | 9.3% | Entire pregnancy | PM2.5 | No | Yes |
| Lee et al., 2013 | 1997-2002 | Pennsylvania, USA | Retrospective cohort study | 34,705 | SGA | 8.5% | Trimester 1 | PM2.5, PM10 | No | Yes |
| Sathyanarayana et al., 2013 | 1997-2005 | USA | Retrospective cohort study | 298,835 | SGA | 10.2% | Trimester 1, trimester 3 | PM2.5 | Yes | Yes |
| Salihu et al., 2012 | 2000-2007 | USA | Retrospective cohort study | 12,356 | SGA | 12.9% | Entire pregnancy | PM2.5, PM10 | No | Yes |
| Le et al., 2012 | 1990-2001 | Michigan, USA | Retrospective cohort study | 24,248 | SGA | 8.3% | Trimesters | PM10 | No | Yes |
| Madsen et al., 2010 | 1999-2002 | Norway | Retrospective cohort study | 25,229 | SGA | 9.6% | Entire pregnancy | PM2.5, PM10 | No | Yes |
| Brauer et al., 2008 | 1999-2002 | Canada | Retrospective cohort study | 70,249 | SGA | 9.0% | Entire pregnancy | PM2.5, PM10 | Yes | Yes |
| Liu et al., 2007 | 1985-2000 | Canada | Retrospective cohort study | 386,202 | IUGR | 10.9% | Trimesters | PM2.5 | No | No |
| Hansen et al., 2007 | 2000-2003 | Australia | Retrospective cohort study | 26,617 | SGA | 7.1% | Trimesters | PM10 | Yes | No |
| Kim et al., 2007 | 2001-2004 | Seoul, Korea | Prospective cohort study | 1,514 | IUGR | 0.9% | Trimesters | PM10 | No | No |
| ***Household air pollution*** | | | | | | | | | | |
| Islam, et al. 2020 | 2015-2016 | India | Cross-sectional study | 80,070 | HAZ | 38.7% | Children < 5 year old | Use of polluted cooking fuel | Yes | No |
| Lamichhane, et al. 2019 | 2006, 2011, 2016 | Nepal | Cross-sectional study | 9,914 | HAZ | 45.0% | Children < 5 year old | Use of polluting fuel | Yes | Yes |
| Amare, et al. 2019 | 2016 | Ethiopia | Cross-sectional study | 3,579 | HAZ | 38.0% | Post-pregnancy (0-59 months) | Traditional vs modern fuel | Yes | No |
| Kim, et al. 2017 | 2005-2014 | South Asia (pooled) | Cross-sectional study | 18,586 | HAZ | 38.40% | Post-pregnancy (6-23 months) | Use of solid fuel | Yes | No |
| Dadras, et al. 2017 | 2011 | Nepal | Cross-sectional study | 900 | HAZ | 39.8% | Post-pregnancy (0-59 months) | Unclean vs cleaned cooking | Yes | No |
| Machisa, et al. 2013 | 2006-2007 | Swaziland | Cross-sectional study | 1,150 | HAZ | 27.60% | Post-pregnancy (6-36 months) | Use of biomass fuel | Yes | No |
| Fenske, et al. 2013 | 2005-2006 | India | Cross-sectional study | 12,176 | HAZ | 36.8%; 17.1% | Post-pregnancy (0-24 months) | Use of diff cooking fuels | No | No |
| Rohner, et al. 2013 | 2010 | Philippines | Cross-sectional study | 477 | HAZ | 26.8% | Post-pregnancy (6-23 months) | Unclean vs cleaned cooking | Yes | No |
| Tielsch, et al. 2009 | 1998-2000 | India | Prospective cohort study | 10,437 | HAZ; SGA | 89.4%; 57.5% | Post-pregnancy (0-6 months) | Use of wood/dung fuel | No | Yes |
| Kyu, et al. 2009 | 2005-2007 | Cambodia, DR, Haiti, Jordan, Moldova, Namibia, Nepal | Cross-sectional study | 28,439 | HAZ | 14.8%; 8% | Post-pregnancy (0-59 months) | Exposure to biofuel smoke | Yes | No |
| Hong, et al. 2006 | 2004 | Bangladesh | Cross-sectional study | 2,570 | HAZ | 43.0% | Post-pregnancy (0-59 months) | Unclean vs cleaned cooking | Yes | No |

**Figure S2.** Pooled estimates of the effect on SGA (all outcome definitions) associated with a 10 μg/m^3^ increase in ambient PM_2.5_ concentration during entire pregnancy


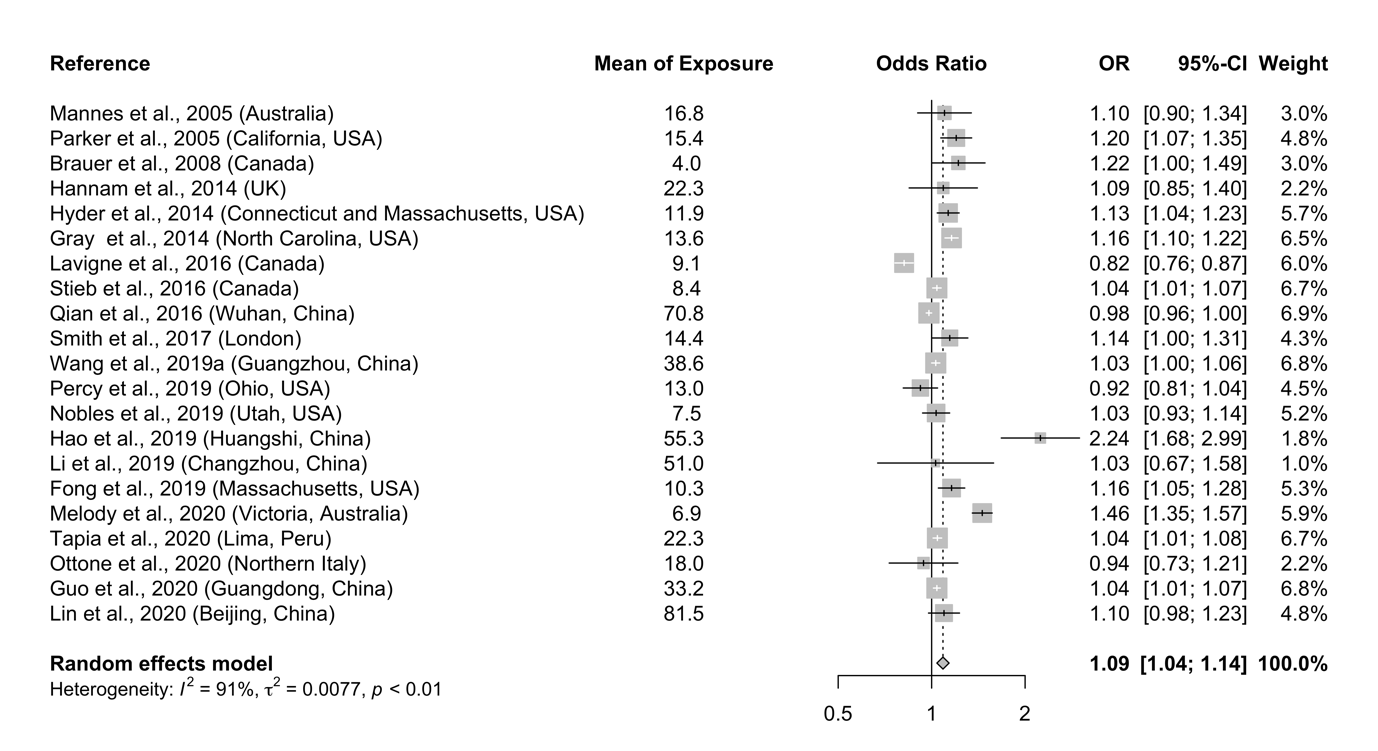


**Figure S3.** Pooled estimates of the effect on SGA associated with a 10 μg/m^3^ increase in PM_10_ concentration during entire pregnancy and three trimesters


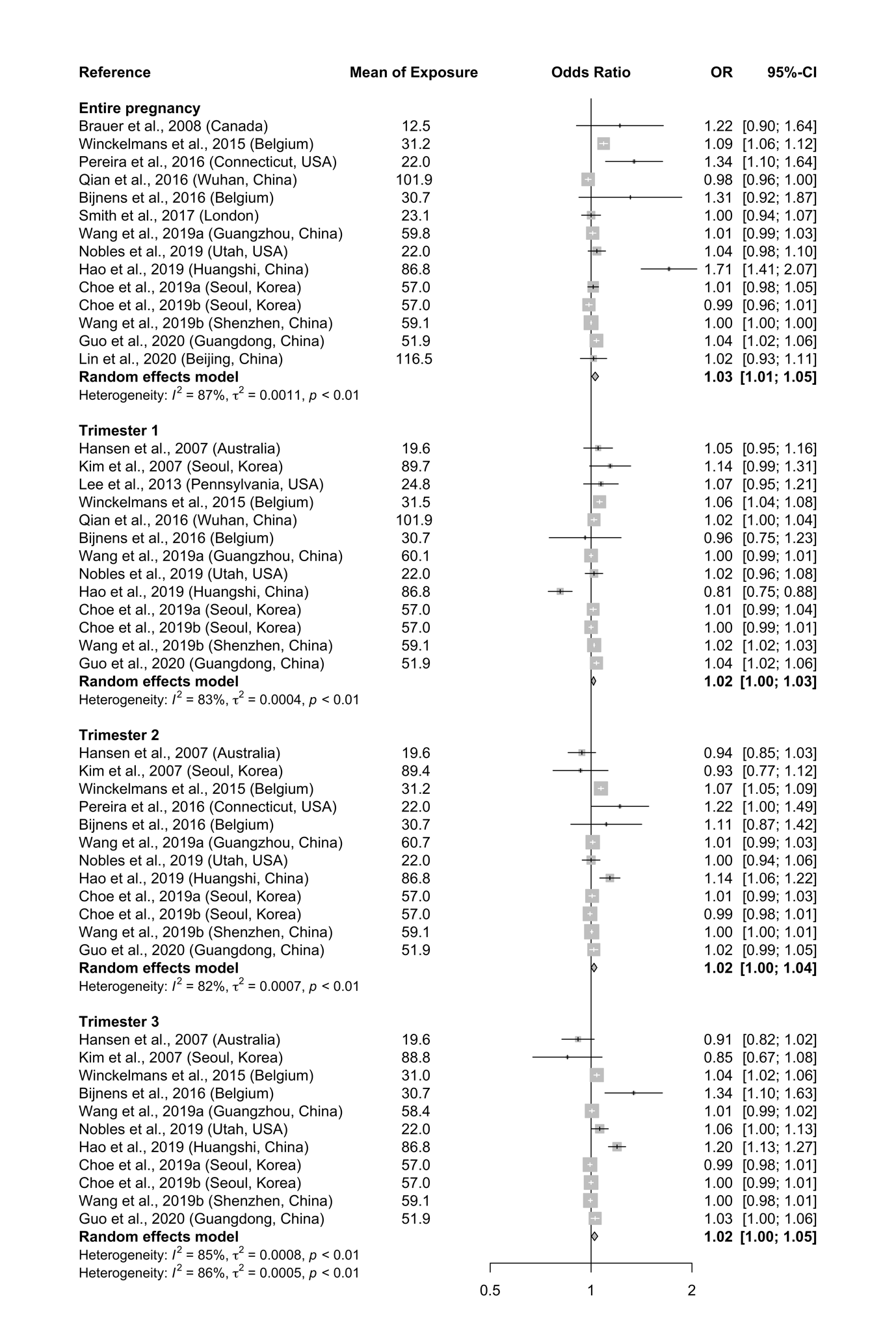


**Figure S4.** Pooled estimates of the effect on severe stunting (HAZ <-3) associated with exposure to household air pollution from solid fuel use compared with cleaner fuels


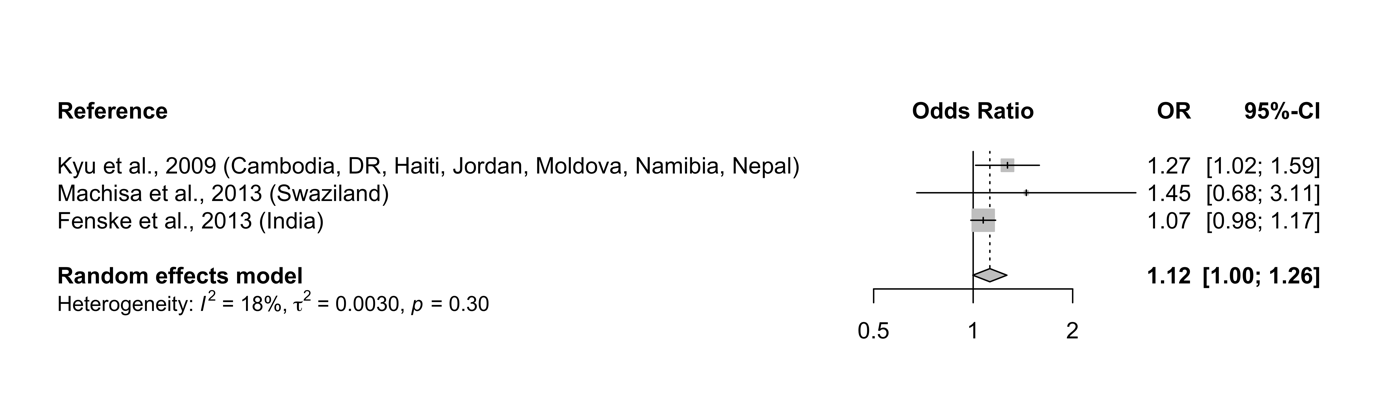

Supplement: Supplementary file 1 — (DOCX 1.06 mb) [file 11356_2021_13719_MOESM1_ESM.docx]
